# Hyaluronic Acid within Self-Assembling Nanoparticles: Endless Possibilities for Targeted Cancer Therapy

**DOI:** 10.3390/nano12162851

**Published:** 2022-08-18

**Authors:** Manuela Curcio, Orazio Vittorio, Jessica Lilian Bell, Francesca Iemma, Fiore Pasquale Nicoletta, Giuseppe Cirillo

**Affiliations:** 1 Department of Pharmacy Health and Nutritional Science, University of Calabria, 87036 Rende, Italy; 2 Children’s Cancer Institute, Lowy Cancer Research Centre, University of New South Wales, Sidney, NSW 2052, Australia; 3 School of Women’s and Children’s Health, University of New South Wales, Kensington, NSW 2052, Australia; 4 ARC Centre of Excellence in Convergent Bio-Nano Science and Technology, Australian Centre for NanoMedicine, University of New South Wales, Kensington, NSW 2052, Australia

**Keywords:** cancer, drug delivery, drug targeting, hyaluronic acid, self-assembling nanoparticles

## Abstract

Self-assembling nanoparticles (SANPs) based on hyaluronic acid (HA) represent unique tools in cancer therapy because they combine the HA targeting activity towards cancer cells with the advantageous features of the self-assembling nanosystems, i.e., chemical versatility and ease of preparation and scalability. This review describes the key outcomes arising from the combination of HA and SANPs, focusing on nanomaterials where HA and/or HA-derivatives are inserted within the self-assembling nanostructure. We elucidate the different HA derivatization strategies proposed for this scope, as well as the preparation methods used for the fabrication of the delivery device. After showing the biological results in the employed in vivo and in vitro models, we discussed the pros and cons of each nanosystem, opening a discussion on which approach represents the most promising strategy for further investigation and effective therapeutic protocol development.

## 1. Introduction

Over the last decade, the application of nanotechnology gained enormous interest as an interdisciplinary approach for cancer theranostics, with the number of researchers focusing on the development of tumor-targeting nanoparticles growing exponentially [1,2].

The unique properties of the nanoparticle system, including proper size, prolonged serum half-life, and specific cell targeting, together with the peculiar features of the tumor site, i.e., leakage of lymphatic drainage, angiogenesis, and increased vascular permeability, enable enhanced molecule accumulation at the tumor site (Enhanced Permeability and Retention effect—EPR) [3]. This offers solutions for both early-stage diagnosis and efficient delivery of therapeutic agents [4], boosting antitumor effects with reduction or reversal of multidrug resistance [5].

The biological performance of any nanosystem is strictly related to its chemical composition and fabrication method, as well as its architecture [6]. The chemical composition can move from organic to inorganic [7], from polymeric and lipid to hybrid and composite materials [8]. Whereas the fabrication method and the purification steps in particular can affect the matrix-associated toxicity, because of contamination with reaction by-products, residual solvents, and un-reacted species [9]. Finally, considering the architecture, non-spherical (e.g., tubes, cubes, cones) and spherical nanosystems can be distinguished, with further classifications possible regarding micellar, vesicular, solid nanoparticle or pristine, layered, and core-shell structures [10]. 

Generally, self-assembly refers to a process in which molecular building blocks (small- or macromolecules, nanomaterials) spontaneously organize into ordered structures with a certain geometric arrangement through local non-covalent interactions [11]. Self-assembly nanotechnologies play a pivotal role in nanomedicine since they are inspired by well-known biological processes, including the formation of the DNA double-helix and the arrangement of phospholipids in cell membranes [12]. The easy scalability of self-assembled nanoparticles (SANPs) preparation methods, which often involve green and inexpensive steps, fits well with the requirements needed for approval from regulatory agencies (e.g., FDA and EMA), thus allowing desirable laboratory-to-clinic-to-industry translations [13]. Moreover, the therapeutic outcomes of SANPs benefit from the ability to encapsulate (or co-encapsulate) with high-efficiency drugs with different physicochemical properties (e.g., hydrophilic, hydrophobic, amphiphilic, and ionic) [14,15], and form the possibility of easy modification with site-specific functionalities. This includes stimuli-responsive groups and small or large targeting moieties [16,17], and takes advantage of the peculiar structural and molecular anomalies at the tumor site (e.g., acidic interstitial pH, altered redox state due to increased cellular metabolism, enhanced oxygen perfusion) [18,19], as well as from the presence of overexpressed receptors for molecular components (e.g., growth factor, interleukins, transferrin) assisting tumor development and metastasis [20]. CD44, transmembrane glycoproteins involved in adhesion, aggregation, migration, and signal transduction, are representative biomarkers for cancer early-stage diagnosis and clinical management [21]. These receptors show a higher affinity for different extracellular elements, including hyaluronic acid (HA), a negatively charged, non-sulfated glycosaminoglycan consisting of D-glucuronic acid and N-acetyl-D-glucosamine repeating units bound by beta-linkages [22]. Thus, the insertion of HA moieties within nanoparticle formulations is a successful strategy for cancer targeting [23], although the choice of HA molecular significantly affects the targeting efficiency [24]. HA with different molecular weights, indeed, possess not only diverse biological functions [25], but also different cell uptake tendencies [26]. Several studies involving nanoparticle systems of different nature, from inorganic nanoparticles to liposomes [27], proved that high molecular HA is a more effective targeting element than low molecular weight HA (e.g., 31 kDa HA was better internalized by HeLa cells than 6 kDA HA [28]), although this is not a general statement since there are experimental evidences that in photodynamic therapy (PDT) protocols, 20 kDa HA exerted better efficacy than 50 kDa or 100 kDa HA [29]. Moreover, by virtue of the presence of hydroxyl, carboxylic, and N-acetyl groups, allowing easy chemical derivatizations, HA can be used in either native or modified forms [30].

Within this review, we overview the impact of HA-SANPs in cancer diagnosis and therapy over the last decade, focusing on the key peculiarities of each formulation. By discussing the chemical composition, the preparation methods, and the biological performances, we aim to highlight the pros and cons of the different proposed approaches, offering a multidisciplinary point of discussion for scientists working in cancer-related research areas. Finally, with a glance to the future in the field, we provide a critical analysis concerning the flaws to be considered and solved for effective bench-to-clinic translation.

## 2. Self-Assembling Nanoparticles Containing Hyaluronic Acid

SANPs are obtained through a process involving the spontaneous organization or aggregation of small molecules, macromolecules, or nanoparticles into stable structures. The interaction forces consist of hydrophobic interactions, π–π aromatic stacking, electrostatic forces, van der Waals forces, and hydrogen bonding [31]. Commonly, hydrophobic interactions drive the formation of SANPs (e.g., micelles or vesicles) composed of amphiphilic molecules where saturated or unsaturated hydrocarbon chain and polar ionic or non-ionic moieties are the lipophilic and hydrophilic counterparts, respectively [32]. In detail, micelles are nanosized particles consisting of a hydrophobic inner portion surrounded by a hydrophilic outer surface [33], while vesicles are hollow structures with an aqueous core surrounded by one or more bilayered membrane [34]. On the other hand, oppositely charged molecules and polymers can self-assembly via electrostatic forces and hydrogen bonding carrying out to nanoplexes and solid nanoparticles [35,36].

The formation of HA-SANPs results from the formation of either electrostatic forces due to their anionic nature or hydrophobic interactions when functionalized with lipophilic moieties. Moreover, supramolecular structures can be obtained when cyclodextrins (CD) and their inclusion counterparts are involved in the self-assembly process [37]. The choice of the suitable preparation technique is driven by the physicochemical properties of the selected HA-based material, as well as by the nature of the interactions between the HA binding blocks and with the loaded therapeutics [38]. Such techniques mainly involved the simple dispersion in water media, with sonication or ultra-sonication methods used as formation co-adjuvants [39], while dialysis processes are used when the HA-derivative needs to be dispersed in organic solvents [40]. The first methodologies can be used when hydrophilic therapeutic agents are used, while hydrophobic molecules should be treated with the second approach [41]. Moreover, typical thin-film hydration or emulsion methods are employed when HA derivatives are organized in liposomal-like structures able to load water-soluble and insoluble bioactive molecules [42,43]. Finally, HA or HA derivatives can be used for the coating (either electrostatic or covalent) of pre-formed SANPs to enhance the targeting behavior [44], but these materials do not fall within the scope of the present review. Here the discussion of the HA-SANPs proposed in the literature for cancer treatment is organized into four main sections, depending on the driving force of the self-assembly process, with further sectioning in native or modified HA.

## 3. Application of HA-SANPs in Cancer Therapy

**Figure 1:**
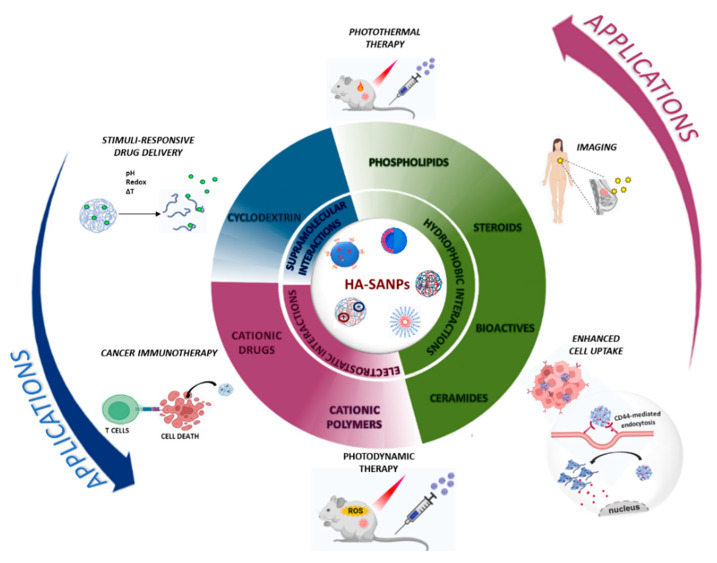
Applications of HA-SANPs for cancer theranostics: indication of the main mechanisms of HA-SANPs formation and the most representative derivatization agents.

As discussed in the previous section, HA-SANPs can be obtained by both electrostatic and hydrophobic interaction forces. In the following paragraphs, we will discuss the main outcome of each approach in cancer theranostics (Figure 1), highlighting the need for HA derivatization to favor the self-assembly process.

### 3.1. HA-SANPs Obtained by Electrostatic Interactions

Table 1 collects the most relevant examples of HA-SANPs by electrostatic interactions. 

The negative charge of HA can be exploited for the formation of nanoparticle structures with cationic drugs such as cisplatin (CDDP) acting as ionic crosslinker [45], with the further insertion of therapeutic agents such as Sorafenib (SRF) [46], Gefitinib (GFT) and Methotrexate (MTX) [47] found to be a valuable strategy for an effective multidrug therapy.

The formation of hydrogen bonds (SRF) or π-π stacking (GFT and MTX) interactions, indeed, enhances the stability of the nanoformulation, improving the in vitro and in vivo pharmacological outcomes by virtue of both a pH-controlled release and a selective CD44 targeting and biodistribution. Following a similar approach, micelle nanocarriers were developed by complexing ferrocene cyclopalladated compound (FCP) with HA in the presence of 5,10,15,20-Tetrakis(4-aminophenyl)-porphin (Tph) as a photosensitizer [48]. The obtained HA-SANPs were successfully employed in a photodynamic therapy (PDT) protocol for the treatment of breast cancer models in vivo.

**Figure 2:**
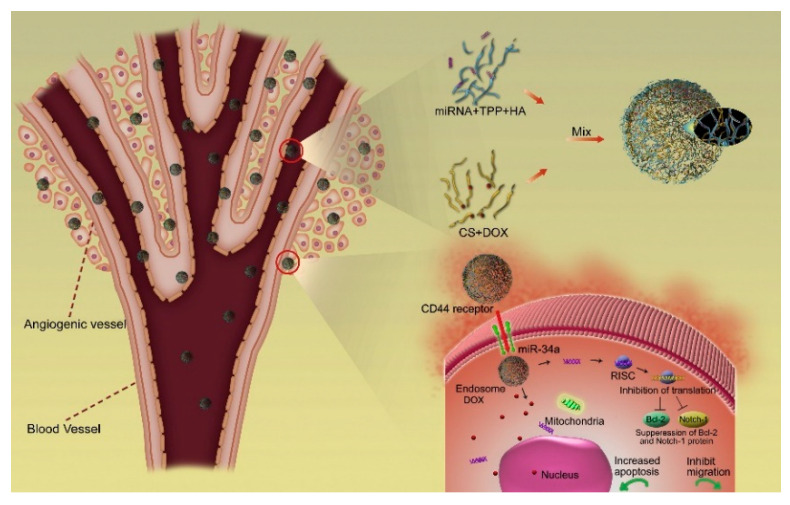
Schematic representation of HA-SANPs obtained from electrostatic interactions between HA and CS for DOX and miR-34a co-delivery. Reprinted with permission from Ref. [50]. 2014, Elsevier Ltd.

HA-SANPs containing native HA were also proposed for the vectorization of genic materials with high efficiency. In these formulations, cationic macromolecules such as Protamine sulfate (PRTS) [49] or Chitosan (CS) [50] were inserted as complexing agents for a miR-34a mimic to improve the loading efficiency, while the presence of HA guaranteed the targeting of CD44 positive cells. Moreover, when CS was used, nanogel systems can be obtained by adding Tripolyphosphate (TPP) in the reaction feed exploiting the well-known ability of such polyanion to act as a crosslinker upon interaction with the ${\mathrm{NH}}_{3}^{+}$ groups on CS side chains [50]. The resulting nanosystem was found to be suitable for dual therapy where Doxorubicin (DOX) was used as a conventional cytotoxic agent in combination with a miR-34a mimic (Figure 2).

SANPs can be also obtained by the direct ionic interaction between HA and CS, with further stabilization of the nanoparticle structure being achieved by oxidation of thiol groups inserted on HA side chains and the formation of disulfide bridges. 

HA/CS complexation was used for the vectorization of Curcumin (CUR) to colon cancer cells upon inclusion in a cyclodextrin derivative [51], while disulfide stabilization was proposed by Xia et al. [52] to prepare dual responsive (pH and redox) DOX delivery systems for the treatment of breast cancer cells. In the latter case, the key advantage of the proposed nanosystem is that, by selecting a proper HA to CS ratio, negative or positive surface charges can be obtained in order to optimize the interaction with ionic drugs. Moreover, targeted chimeric nanocarriers for the co-delivery of DOX and siRNA were constructed by conjugating two different HA-SANPs through redox-sensitive thiol–disulfide bonds. HA-SH was combined to N,O-Carboxymethyl chitosan (NOCC) for DOX loading, while the Calcium Phosphate siRNA complex was encapsulated within Dopamine (DA)-HA-SH based SANPs [53]. 

HA derivatization with SH groups was also proposed for the formation of an oligoDNA complex able to self-assemble in a K^+^-dependent manner in a G-quadruplex causing selective cancer cell blebbing and death [54]. 

Metal chelation is another methodology useful for promoting the self-assembly of HA nanoparticles, with calcium ions being widely explored as pH-responsive crosslinking agents [55]. On the other hand, Mn^2+^ ions are capable of both stabilizing SANPs by forming crosslinks and acting as magnetic imaging agents. Moreover, by competitive coordination, manganese ions are able to decrease the glutathione (GSH) intracellular concentration with a beneficial effect on PDT protocols. These findings were recorded in a work by Pan et al., where multifunctional HA-SANPs for combined chemo-photodynamic therapy were developed taking advantage of the loading of DOX as an antineoplastic agent, and Chlorin e6 as PDT agent, as well as from the HA derivatization with Histidine (His) residues to enhance the affinity towards Mn^2+^ ions [56]. Pt ions within CDDP molecules can also act as metal crosslinkers by ligand exchange between the NH_2_ and the hydrazide groups of HA-3,3′-dithiobis(propionohydrazide) derivative (HHA) within HHA/BSA nanoparticles. Moreover, the simultaneous coordination between CDDP and the sulfonic groups of ICG allowed the formation of SANPs for dual chemo-photothermal therapy [57].

The ionic nature of HA can be responsible for the formation of strong hydrogen bonding with different species, including water-insoluble compounds of biological interest. In this regard, HA was proposed as a targeting dispersant agent for the immunostimulatory monophosphoryl lipid A (MPL) in combination with the extract from the bark of the *Quillaja saponaria Molina* tree (QS-21) or Imiquimod (R837). The resulting complexes were found to enhance both humoral and cellular immunity and thus can be used as a vaccine system (Ovalbumin—OVA as model antigen) to induce prophylactic anticancer immune response preventing tumor recurrence and growth in vivo [58].

### 3.2. HA-SANPs Obtained by Hydrophobic Interactions

The HA functionalization with lipophilic moieties was proved as a valuable strategy to confer amphiphilic properties allowing the organization of HA-derivative in stable nanoparticle systems. Different molecular specimens can be used as lipophilic moieties, which are here classified in three main classes, namely the steroid-, lipid-, and phenyl- based structures.

#### 3.2.1. Steroid Modified HA in the Formation of HA-SANPs 

HA backbone was hydrophobically modified by conjugation with cholesterol (CHL) moieties via carbodiimide chemistry to form either nanoparticle structures for drug and gene delivery or liposomes when inserted in a proper mixture of phospholipids (Table 2).

Taking into consideration that HA-based nanoparticles cannot directly encapsulate anionic siRNA molecules due to the net negative charge, Choi et al. proposed the incorporation of siRNA/2b protein complexes into HA-CHL nanoparticles. They found that the nanosystem was able to selectively deliver the 2b protein/siRNA complexes to melanoma cells with up-regulated CD44 receptors, release the siRNA within the endocytic compartments due to dissociation of the 2b protein/siRNA at acidic pH, and effectively suppress the expression of the target gene [59]. HA-CHL nanoparticles were also endowed with redox responsivity when a GSH-sensitive linker such as Cystamine (cys) was used to connect HA and CHL molecules. By this strategy, DOX and IR780, as cytotoxic drugs or photosensitizing agents, respectively, were vectorized to breast cancer cells in both in vitro and in vivo models. GE11 was used as a targeting peptide to improve the selectivity of the DOX release [60], while cell death occurred by high ROS generation upon IR780 laser irradiation (PDT step) followed by high increased temperature (photothermal effect—PTT) [61].

The insertion of HA-CHL conjugate in liposome formulations can be performed by either post-insertion in pre-formed vesicular formulation or hydration of the thin layer film with an HA-derivative solution. By the post-insertion method, hydrogenated Lecithin-based liposomes for DOX and Paclitaxel (PTX) combined therapy were prepared, with the HA residues on the outer surface able to discriminate between CD44+ (breast cancer) and CD44− (fibroblast and liver cancer) cells [62]. On the other hand, complex liposomal structures can be obtained when HA-CHL aqueous solution was employed as a hydrating agent. The efficiency of such a system was further enhanced by conjugation with the Lys-Leu-Val-Phe-Phe (KLVFF) peptide, a key sequence involved in the β-sheet fibril formation showing antimetastatic activity [63].

**Figure 3:**
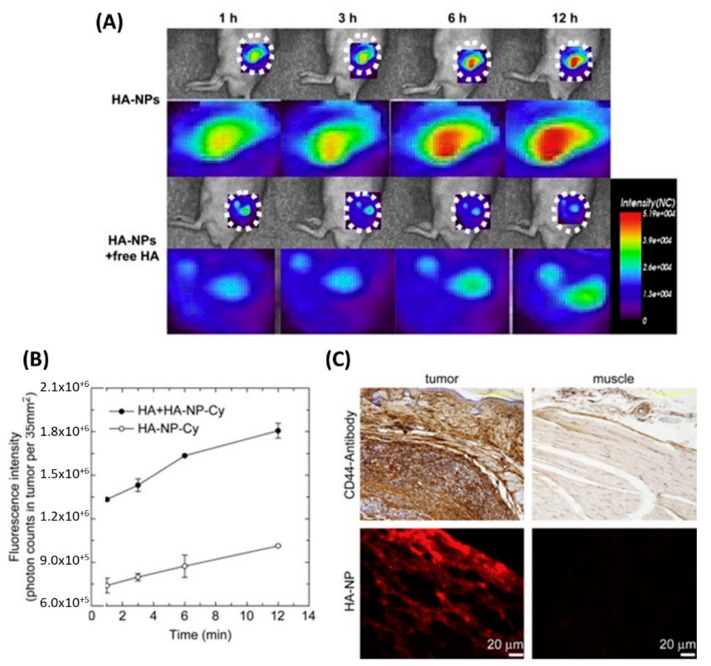
(**A**) In vivo fluorescence images of self-assembled HA-5-βCA nanoparticles and (**B**) their quantification in tumor-bearing mice with and without pre-injection of free-HA. (**C**) Magnified images of tumor and muscle tissues. Adapted with permission from Ref. [66]. 2009, Elsevier Ltd.

Together with CHL, other steroid structures such as testosterone (TST) [64], 5-β-Cholanic acid (5-βCA), and Deoxycholic acid (DOCA) have been proposed for the lipidization of HA. Cyanine-labeled self-assembled HA-5-βCA nanoparticles (Figure 3), prepared via different techniques, were effectively targeted in different cancer cells and tissues, including breast, prostate, and squamous carcinomas [65,66].

Such nanosystems were proposed for the delivery of PTX [67] with the further possibility to modulate the release by exploiting the hydrolytic activity of Hyaluronidase (HAase) selectively expressed within the tumor cells [68]. Moreover, the encapsulation of Perfluoropentane (PFP) within HA-5-βCA SANPs allowed the obtainment of echogenic materials for the early-stage diagnosis of colon cancer [69]. 

The effective accumulation of HA-SANPs into the tumor site is a result of a combined EPR and active targeting. Nevertheless, the high affinity of HA towards the HA receptor (HARE) expressed by liver sinusoidal endothelial cells can determine high liver uptake, with a possible reduction of the therapeutic efficiency. The PEGylation of HA-SANPs can be a valuable approach to specifically address this issue: PEG molecules, indeed, form a hydrophilic shell on the nanoparticle outer surface, conferring stealth properties towards the phagocytic cells of the reticuloendothelial system and thus prolonging the blood circulation time. When applied to self-assembled HA-5-βCA nanoparticles, the selective biodistribution of nanocarriers [70] allowed the pharmacokinetics profiles of different chemotherapeutic agents, such as DOX, CPT [71], and Irinotecan [72] to be improved.

The targeting efficiency of HA-SANPs can be enhanced by inserting pH and/or redox responsive functionalities in the nanoparticle structure. For this purpose, the imidazole ring of His (pH responsivity) [73] and the disulfide bridges of cys (GSH responsivity) [74] were conjugated to HA-DOCA derivatives, obtaining PTX vectorization to breast cancer both in vitro and in vivo. 

As a further development of these systems, the HA-DOCA derivative was conjugated or co-formulated with PEG and Pluronic (PF 127) species, obtaining nanocarriers able to modulate the release of DOX and PTX in response to the acidic and GSH-rich tumor environment. In detail, the PEGylation processes allowed the enhancement of the biodistribution profiles [75], while the presence of PF 127 was used to improve cellular uptake thus counteracting the insurgence of multidrug resistance processes [76].

#### 3.2.2. Lipid-modified HA in the formation of HA-SANPs 

The introduction of lipophilic moieties on the HA backbone can be reached by conjugation with lipid chains, belonging to phospholipids, ceramides, fatty acids, amines, and alcohols (Table 3).

Phospholipids are highly biocompatible compounds widely employed for the fabrication of different drug delivery systems, such as micelles, liposomes, solid lipid nanoparticles, micro- and nano-emulsions [95]. They can serve as HA lipidizing agents, allowing the obtainment of SANPs with different architectures [77] for the vectorization of cytotoxic drugs and gene to pancreatic [78] and lung [79] carcinomas. In the latter case, the insertion of the transferrin (Tf) motif within the nanoparticle formulation enhanced the targeting behavior and the transfection efficiency which was found to be significantly superior to that of conventional liposomes used as a control.

Ceramides (CE) belong to the sphingolipids group and consist of an acylated long-chain sphingosine base. Although they have a positive net charge, ceramides are used as structural components of nanoformulations by virtue of their ability to easily move across cell membranes [96]. 

**Figure 4:**
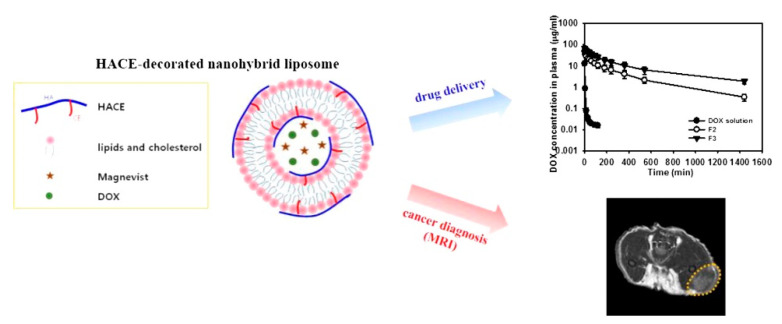
Self-assembled HA-CE nanoparticles for cancer imaging and DOX delivery. Reprinted with permission from Ref. [82]. 2013, Elsevier B.V.

HA-CE conjugates, alone or in combination with phospholipids and pluronics, were properly used as a component of nanoparticle, liposome, and micelle formulations. Pure HA-CE nanoparticles were tested as a vehicle for the vectorization of PTX in combination with Hypocrellin B (HB) as a photosensitizer in synergistic chemo- and photodynamic- treatment of lung cancer [80]. The choice of cationic or neutral phospholipids allowed the insertion of HA-CE in liposomal bilayer for the delivery of plasmid DNA [81] or drug to MDA-MB-231 breast cancer cells, with the possibility to simultaneously load a gadolinium derivative for Magnetic Resonance Imaging (MRI) [82] (Figure 4).

When Pluronic 85 was combined with HA-CE, a micellar drug formulation able to reverse the Docetaxel (DTX) resistance in MCF-7/ADR xenograft mice was obtained [83].

Fatty acid derivatives, including amines and alcohols, represent potentially one of the most useful types of lipidizing agents for macromolecules of biological interest, with the amphiphilic behavior being able not only to promote self-assembling processes in physiological environments but also to module the biological properties of the resulting conjugate [97]. The HA derivatization with Dodecylamine (DDA) residues was used for the obtainment of micelle nanocarriers for the delivery of DOX, and the targeting behavior was further enhanced by inserting pH-responsive His residues [84]. HA-DDA derivative was also co-formulated with different surfactants to improve the intracellular delivery of DTX in lung cancer cells [85], while the insertion of HA-hexadecylamine (HA-HDA) into a liposome architecture was proposed as a strategy to co-encapsulate DTX and Iron Oxide Magnetic Nanoparticles (IONPs) in a combined chemo- and photothermal therapeutic nanoplatform [86]. HA-HDA conjugate was also combined with Poly(lactic-co-glycolic acid) (PLGA) in a O/W emulsion process for the preparation of SANPs for PTT by encapsulation of a Zn-phthalocyanine (PHC) complex [87]. By conjugation of the hydrophobic unit of Dioleic acid (DO) to the carboxyl group of HA by carbodiimide chemistry, Xu and co-workers developed hyalurosomes for the targeted delivery of Indocyanine green (ICG) into lung tumor cells, where it exerts PTT and PDT functions [88]. Moreover, as discussed for steroid-modified HA, HA-SANPs based on HA-fatty acid derivatives were endowed with redox responsivity by insertion of cys [89] or alkanethiol [90] residues and further engineered by PEGylation of the outer surface [91]. 

Finally, an original approach for the lipidization of HA backbone was proposed by the Chen research group, that synthesized amphiphilic and pH-sensitive HA-acetal-menthone (MGK) derivatives able to self-assembly in micelle systems for the vectorization of CUR to squamous carcinoma [92] and mesothelioma [93], with Folic acid (FA) used as dual targeting element for breast and lung cancer cells [94].

#### 3.2.3. Phenyl Compounds-Modified HA in the Formation of HA-SANPs 

Aromatic compounds were also employed as HA lipidizing agents, due to the enhanced loading capacity of hydrophobic bioactive agents via π-π stacking (Table 4). 

Aminopropyl-1-pyrenebutanamide (PBA) was found to enhance the loading and selective biodistribution of ICG [98] and Orlistat (ORL) [99], an FDA-approved inhibitor of fatty acid synthase. It was observed an enhanced ORL activity not only in pancreatic cancer cells, the main target of this lipophilic drug but also in breast cancer cells overexpressing CD44 receptors, confirming the high internalization efficiency of HA targeted SANPs. 

Another approach involved the HA derivatization with 2,3,5-Triiodobenzoic acid (TIBA), a contrasting agent for X-ray computed tomography [100], allowing the preparation of HA-SANPs that, upon DOX loading, were successfully applied in the treatment of squamous cell carcinoma. The same cancer model was employed to test the in vitro and in vivo efficiency of hybrid nanoparticles consisting of hydrophobic IONPs linked to HA through Dopamine (DA) spacer [101]. Here, Homocamptothecin (HCPT) acted as a cytotoxic agent, while the magnetic properties of IONPs were used for both targeting and imaging applications.

Finally, by virtue of the high efficiency of pH/redox responsive SANPs, cys residues were used as a spacer between HA and the hydrophobic Tetraphenylethylene (TPE) moieties for the preparation of micelles able to selectively vectorize DOX to cervix and ovary cancers [102]. Disulfide-containing HA-SANPs were also obtained by oxidizing an HA-cysteine derivative with 6-Mercaptopurine (MP), with the resulting micelle being destabilized in the tumor micro-environment allowing a selective release of the loaded anticancer drug [103].

### 3.3. HA-SANPs Obtained by HA Modification with Polymeric Materials

Polymeric SANPs have been widely demonstrated as safe and powerful anticancer nanocarriers due to their high chemical versatility and the possibility to easily tailor the physicochemical properties, the permeability, and thus the kinetics of drug release. HA was used as a targeting motif of the self-assembling polymeric conjugate of both natural and synthetic origin (Table 5). 

**Figure 5:**
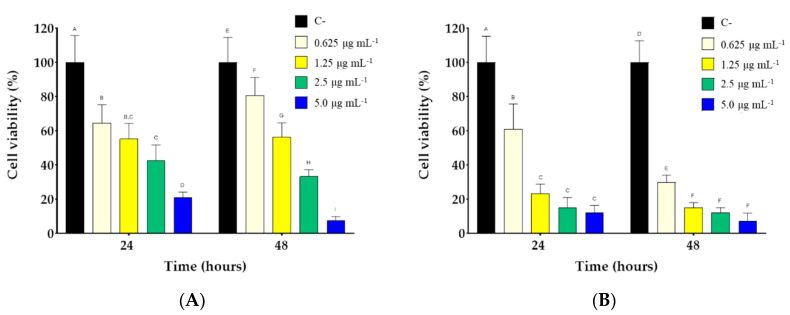
Cell viability of BALB/3T3 (**A**) and MDA-MB231 cells (**B**) treated with DOX@HA-SANPs (drug concentration from 0 to 5 µg/mL) after 24 and 48 h. Within each group, different letters denote statistical differences for *p* < 0.05, n = 5. Reprinted from Ref. [105].

HA was conjugated to serum albumins because of their intrinsic ability to bind and transport biomolecules through the blood circulation [134], allowing the preparation SANPs for PTX, Imidazoacridinones (IA) [104], and DOX [105] vectorization. In the case of DOX vehicles, a cys linker was also inserted between HA and protein for conferring GSH responsivity (Figure 5).

HA-polypeptides, including Poly(γ-benzyl-L-glutamate) (PBLG), Poly(N-ε-carbobenzyloxy-L-lysine) (PZLL), and Poly(diisopropylaminoethyl) aspartamide (PIPASP) were used as building blocks of HA-SANPs. PBLG and PZLL allow the self-assembly by virtue of their highly ordered α-helix secondary structure [106,107,108], and hydrophobic behavior [109], respectively, while PDIPASP acts as a pH-responsive moiety [110]. 

Owing to their high biocompatibility and biodegradability, Poly(lactic acid) (PLA), Poly(glycolic acid) (PGA), and their copolymers (PLGA) have been extensively investigated for the preparation of highly engineered nanocarriers [135]. PLA/PLGA moieties were conjugated to HA to serve as hydrophobic counterparts needed to form robust self-assembling nanostructures in aqueous media, and effectively vectorize chemotherapeutics such as DOX [111,112], DTX [113], and PDT agents [114] to colon, breast, and lung cancers. As discussed for other typologies of HA-SANPs, also for the HA-PLA/PLGA conjugates the redox responsive approach was widely explored in order to obtain a targeted release of the therapeutic agent in the intracellular space of cancer cells. For this context, Wang et al. developed disulfide-crosslinked HA-SANPs consisting of star PLGA-Lipoic acid (sPLGA-LA) conjugate self-assembled in the presence of HA-PLA conjugate. As a post-formulation crosslinking strategy, LA residues were oxidized by Dithiothreitol (DTT) to ensure the selective vectorization of DTX to lung cancer both in vitro and in vivo as a consequence of SANPs destabilization within the tumor environment [115]. Moreover, HA-PLGA conjugates can be organized in redox-responsive nanoparticle structures by inserting GSH-responsive linkers between HA and PLGA counterparts [116], with the possibility to further enhance the site-specificity of the drug release by the insertion of other targeting elements such as FA [117] and Tf [118]. 

In a more innovative approach, mesenchymal stem cells (MSC)-based “Trojan horse” micelles were proposed as a more selective nanocarrier to overcome non-specific distribution often attributed to the wide expression of CD44 within tissues and organs. In detail, PTX-loaded HA-PLGA SANPs were shielded by endocytosis within MSC micelles for an effective orthotopic glioma therapy [119]. 

Poly(ε-caprolactone) (PCL) is another key polymer widely used in biomedical fields for the preparation of delivery vehicles due to its ability to control the drug release kinetics and not significantly lower the environmental pH upon degradation [136]. SANPs based on HA-PCL conjugate were, indeed, successfully used for the vectorization of chemo- [120] and radio- [121] therapeutics. Moreover, the further insertion of PEG moieties was found to improve the blood circulation time [122], while the derivatization with 2-(Pyridyldithio)-ethylamine (PDA) conferred the possibility to perform a post-crosslinking step in the presence of DTT for enhanced GSH responsivity [123]. 

The targeting properties of HA were combined to the high biocompatibility and chemical versatility of acrylic-based polymers [137], and the resulting polymer conjugate was suitable for the preparation of SANPs with a variety of architecture, including micelles and nanogels. In this regard, redox-responsive micelles for the treatment of squamous [124] and breast carcinomas [125] were developed by self-assembly and post-disulfide crosslinking either in the presence or absence of DTT, while microfluidics and catalyst-free photo-click crosslinking allowed the preparation of nanogels with dual targeting efficiency [126]. Different research groups proposed the synthesis of HA derivatives able to organize in nanogel structures upon reaching a critical assembling temperature, with SANPs for the vectorization of DOX and PTX to breast [127] and ovarian [128] cancers being some key examples of this approach. Moreover, the insertion of photocleavable coumarin moieties allowed the possibility to trigger the PTX release in response to the application of a light stimulus [129].

Finally, when poly(ethyleneimines) (PEI) were used as HA derivatizing agents, targeted gene-delivering SANPs were obtained. Ganesh et al. performed a screening of different NH_2_-containing HA derivatives to assess the siRNA encapsulation efficiency, showing the superior performance of HA-PEI, as well as the possibility to combine these features with redox responsibility and PEG-shielding properties [130]. The same authors proposed a CDDP and siRNA co-therapy for lung cancer treatment: HA-SANPs obtained by the co-assembly of HA-ODA and HA-PEG derivatives were used as CDDP vehicles co-administrated in xenograft mice in combination with siRNA-loaded HA-PEI/HA-PEG nanosystem [131]. Genetic materials were also loaded in star HA derivatives consisting of HA-branched PEI [132] and β-CD branched oligoethylenimine (OEI) [133], allowing effective transfection to melanoma and breast cancers, respectively.

### 3.4. HA-SANPs by Supramolecular Assemblies

CDs are water-soluble, nontoxic, and low-cost cyclic oligosaccharides with six to eight D-glucose units linked by α-1,4-glycosidic bonds, obtained from biodegradation of starch using Glucanotransferase enzyme. They are widely explored for the delivery of bioactive agents by virtue of their ability to selectively host inorganic and/or organic molecules in their hydrophobic cavity. Nevertheless, the use of native or simply-modified CD can be limited due to unfavorable pharmacokinetic profiles [138]. To overcome this disadvantage, multiple CD units were combined in the so-called CD-based supramolecular assemblies, nanoarchitectured materials with several binding sites for substrates mimicking the typical cooperative “multimode, multipoint” binding effect observed in biological systems, thus enhancing the loading efficiency and tailoring the release behavior [139]. 

HA-SANPs involving the formation of supramolecular CD complexes can be obtained by the derivatization of α- and β-CD with either HA or the other components of the nanosystem. Finally, some examples of dual host–guest interactions are listed (Table 6).

CUR/Oxaplatin (OXPt) complex was included in HA-βCD with the formation of supramolecular SANPs for the treatment of pancreatic and lung cancers [140], while ultra-strong host-guest interaction between Permethyl-β-CD (PMCD) and Porphyrin (Ps) was used in the preparation of HA-SANPs for the delivery of PTX-Ps to ovarian cancers cells combining the therapeutic efficiency of PTX and the fluorescence properties of Ps [141]. Moreover, high efficient chemodynamic (CDT) and photodynamic therapy (PDT) protocols for breast and lung cancers were developed when Fc-Cinnamaldehyde (Fc-CA) pH-responsive prodrug and Cucurbit[8]uril photosensitizing derivatives were used as the guest molecule of HA-βCDa and HA-αCD, respectively [142,143]. In a different approach, Liu and co-workers explored the possibility to use HA-αCD derivative as the hosting element for a UV-responsive azobenzene-diphenylalanine compound with a positively charged imidazole group able to coordinate siRNA. UV irradiation triggers the cis-trans isomerization of the azobenzene double bond resulting in an HA-SANPs disassembly and siRNA release [144]. Among the different molecules forming strong inclusion complexes with CD, Adamantine (Ad) was widely used as a derivatizing agent of either guest molecules with improved affinity for HA-CD conjugates, or HA with the aim to confer targeting activity to CD-based SANPs.

Following the first approach, the coordination of Adamaplatin (Ad-Pt) [145] and Ad-CPT redox responsive prodrug [146] were explored as therapeutic tools for the treatment of ovarian cancer and osteosarcoma, respectively, while MRI and near-infrared (NIR) imaging protocols were developed when the host-guest interaction involved diagnostic molecules such as Gadolinium and Cyanine dye derivatives [147]. On the other hand, Ad-HA acted as the guest molecule of CD derivatives for Chlorambucil (CBL) [148] and DOX [149] release, and further improvements were obtained upon conjugation of CD to CPT [150], or PEI [151] to enhance the drug and gene targeting efficiency, respectively. 

Double host-guest interactions due to the presence of CD on both HA and guest molecules were involved in the formation of supramolecular SANPs for Ruthenium-based PDT [152] or for combined chemo-PDT protocol upon derivatization of guesting permethyl-β-CD and hosting HA-βCD with redox responsive CPT and Triphenylphosphine moieties, respectively [153]. 

**Figure 6:**
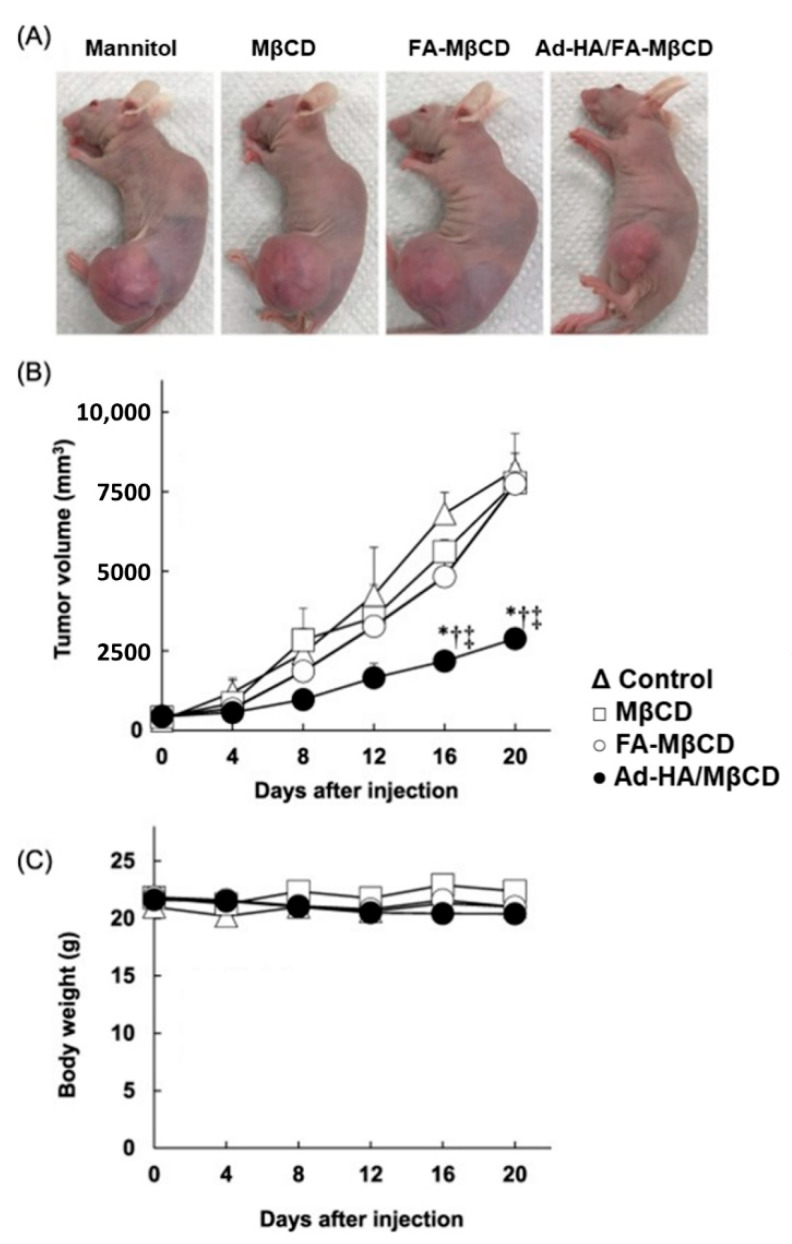
Effects of adamantane-grafted hyaluronic acid/folate-appended methyl-β-cyclodextrin (Ad-HA/FA-MβCD) on tumor growth (**A**,**B**) and body weight (**C**) after an intravenous administration to BALB/c nu/nu mice bearing HCT116 cells. * *p* < 0.05, compared with control (5% mannitol solution). ^†^ *p* < 0.05, compared with MβCyD. ^‡^ *p* < 0.05, compared with FA-MβCyD. Reprinted with permission from Ref. [156]. 2018, Elsevier B.V.

Finally, the specific CHL-binding affinity of Methyl-βCD (MβCD) can be used to extract CHL from the membrane of cancer cells, thus inducing apoptosis, either by the direct conjugation to HA [154] or as a hosting molecule for Ad-HA [155,156] (Figure 6).

### 3.5. HA-Prodrug Nanoassemblies

Although small-molecule cytotoxic drugs remain the mainstream tools for cancer treatment, the narrow therapeutic window and unfavorable pharmacokinetic properties, due to quick clearance and lack of selectivity, significantly hinder their long-term employment in clinical practice thus limiting the therapeutic outcomes [157]. Apart from the strategies involving the encapsulation of bioactive within SANPs extensively discussed in the previous sections, another approach involves the covalent conjugation of these molecules to polymeric materials, with the formation of the so-called polymeric prodrugs, specimens with enhanced water solubility, chemical stability and enhanced permeation within the tumor environment [158,159,160]. 

Moreover, the insertion of proper stimuli-responsive linkages allows the release of the bioactive element to be finely tuned according to the therapeutic needs [161,162]. Polymer prodrugs show the double advantage of high drug loading with negligible formulation-trigged adverse reaction [163], and superior self-assembly ability due to the balancing between the drug to drug (driving self-assembly) and the drug to water (driving dissolution) intermolecular forces [164]. 

The organization in prodrug nano-assembly can be exploited for combination protocols where a second therapeutic agent is loaded within the nanostructure [165]. The most relevant examples of HA-prodrug nanoassemblies are collected in Table 7 and discussed below.

PTX and DTX, the two most representative members of taxane drugs used in clinical practice, were conjugated to HA with the obtainment of prodrug SANPs for the treatment of liver and breast carcinomas. The conjugation strategies involved the condensation via either carbodiimide [166] or TBA chemistry [167], as well as click chemistry [168], while the insertion of tailored spacers between bioactive and HA counterparts confers responsivity to the acidic and GSH-rich tumor environment. Moreover, the presence of HA [167] and the insertion of peptide spacers [169], susceptible to the hydrolytic activity of HAase and proteases overexpressed within the cancer cells, allowed the enhancement of the targeting efficiency due to enzyme-triggered disassembly. Similarly, DOX was introduced as a bioactive hydrophobic moiety of HA-SANPs, reaching the selective pH-sensitive vectorization of the antineoplastic antibiotic [170], while the insertion of disulfide bridges was used as a dual-stimuli responsive strategy for lung [171], liver [172], and breast [173] cancers. 

MTX is another hydrophobic drug used for conferring amphiphilic behavior to HA backbones. Upon conjugation to HA, MTX works as both a cytotoxic agent by inhibiting the Dihydrofolate reductase [174] and targeting moiety because, due to the structural similarity with FA, acts as a ligand for FA receptors overexpressed in many cancer cell types [175]. 

In the attempt to deliver the therapeutic doses of redox-responsive HA-3,3′-dithiodipropionic acid (DTPA)-CPT micelles into tumors, limiting the high liver accumulation [176], Chen et al. proposed the conjugation of HA-DTPA-CPT conjugate to PLA via acid-labile 2-propionic-3-methylmaleic anhydride (CDM) linkers and the subsequent incorporation of micelles into electrospun fibers [177]. The results confirmed the antitumor performance of fiber fragments, as well as the acidic-triggered release of HA-DTPA-CPT from the fibers and the self-assembly of the prodrug in the tumor tissues.

One of the main drawbacks of conventional chemotherapeutic protocols is the insurgence of multidrug resistance (MDR), a complex biological event involving different pathways, such as increased efflux of drugs, restoration of DNA damages, and development of antiapoptotic mechanisms [200,201]. Different strategies have been developed to address this issue [202,203,204], mainly based on the inhibition of P-glycoprotein (P-gp), membrane transporters belonging to the ATP binding cassette family, responsible for drug efflux through an ATP-dependent mechanism [205,206]. Among the different P-gp inhibitors proposed in the literature, D-α-tocopheryl poly(ethylene glycol) succinate (TPGS), coupling the intrinsic biological function with the self-assembling properties, was found to be an ideal nanocarrier for MDR reversal [207]. TPGS was successfully combined with different HA prodrugs, obtaining HA-SANPs with superior anticancer performance. Dasatinib (DAS), a second-generation tyrosine kinase inhibitor, was conjugated to HA and together with TPGS, the resulting nanoassemblies were tested as therapeutic agents [178]. Vitamin E succinate could also be conjugated to HA to create carriers for conventional chemotherapeutics such as DTX [179]. The authors also added TPGS conjugated to a cell-penetrating peptide to enhance the internalization efficiency. Moreover, CUR [180] and rosiglitazone (ROZ) [181] were co-loaded as MDR reversing and adipogenesis agents, respectively. HA- SANPs were also obtained with HA-VES and proposed as nanocarriers for DOX treatment in Adriamycin resistant breast cancer cells [182], as well as for the combined DOX/CUR [183] or DTX/programmed cell death ligand 1 (PD-L1) antibodies (anti-PD-L1) [184] protocols exploiting CUR as coadjuvant and the Anti-PD-L1 as an immune checkpoint.

Polyphenols have been explored as anticancer therapeutics acting via multiple mechanisms, mainly related to the pro-apoptotic effect and modulation of the cell redox balance [208,209,210]. Their application in clinics needs suitable carrier systems to overcome their poor pharmacokinetics, and it was widely accepted that the conjugation to macromolecular systems is a valid approach for improving their stability and bioavailability [211,212]. Polyphenols, such as CUR, Quercetin, and Epigallocatechin-3-O-gallate (EGCG), were used for the synthesis of HA amphiphiles with biological activity. HA-CUR and HA-QC conjugates were explored as functional nanocarriers for the pH-responsive delivery of DOX [185] and DTX [186], respectively, while EGCG was used for enhancing the ability of HA-SANPs to complex CDDP molecules [187] and cytotoxic proteins such as Granzyme B (GzmB) [188]. HA-SANPs for the delivery of PTX to multiple solid tumors were obtained by the self-assembly of HA-Glycyrrhetinic acid (GCA), taking advantage of the GCA anti-inflammatory and immuno-modulating properties, as well as its ability to reverse MDR [189,190].

Bioactive molecules for PTT and PDT such as IR780 [191] and Diiodostyryl bodipy (DB) [192], were also conjugated to HA for the fabrication of HA-SANPs suitable for the treatment of bladder and colon cancers both in vitro and in vivo. A different PDT protocol, developed by Feng et al., is based on the delivery of Ce6 by a nanoplatform consisting of BSA, Cyclopamine (CYC), and HA-SeSe-Ce6 amphiphile [193]. The anticancer efficiency is the result of the synergistic contribution of each component: BA is the base material for tumor residence, CYC disrupts the extracellular matrix (ECM) barrier thus allowing HA-SeSe-Ce6 penetration, HA-SeSe-Ce6 is the PDT agent selectively releasing Ce6 within the tumor cells in response to the GSH concentrations. 

In recent years, Nitric Oxide (NO) donors are emerging as effective anticancer therapeutics able to release NO at the tumor site where it causes tumor regression and metastasis inhibition. Since NO exerts such anticancer activity only at high concentrations, while acting as a pro-carcinogenic agent at low concentrations, it is of key importance to ensure high NO levels at the desired place in the body [213]. HA-SANPs able to generate NO redox reactions catalyzed by intracellular glutathione S-transferase π and to encapsulate DOX in the hydrophobic inner core were developed by derivatizing HA with Diethylamine NONOate (DEA/NO). The resulting material was found to greatly enhance the DOX anticancer efficiency in the treatment of highly aggressive hepatoma cells [194]. An alternative anticancer therapy based on the use of Bovine serum amine oxidase (BSAO) as a bioactive agent was proposed by Montanari et al. [195]. BSAO catalyzes the oxidative deamination of primary amines, such as spermine and spermidine, carrying out the formation of highly cytotoxic aldehyde and hydrogen peroxide. Injectable hydrogels were developed by the self-assembly of HA-CHL-BSAO with the aim to maximize the selectivity of enzymatic activity to melanoma cells. Further extensions of the HA-prodrug nanoassemblies concern the use of Perylene diimide (PDI) [196] and Oligophenylenevinylene (OPV) [197] derivatives as diagnostic tools for the early detection of solid tumors.

**Figure 7:**
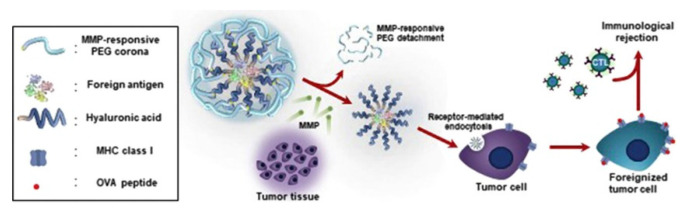
Representation of MMP9 responsive PEGylated HA-OVA targeted cancer immunotherapy. Reprinted with permission from Ref. [199]. 2017, Elsevier B.V.

Finally, HA-OVA conjugates were proposed as targeted delivery systems for pathogen-derived foreign antigens (OVA), determining a robust CD8+ T cell response upon recognition of tumor cells presenting non-self foreign antigens by the host immune system [198]. As a further upgrade of this concept, Shin et al. proposed the use of a matrix metalloproteinase 9 (MMP9) cleavable linker to attach PEG moieties to HA-OVA conjugate [199]. Within the tumor site, the hydrolytic activity of MMP9 allowed the removal of the PEG shell, with the site-specific HA exposure and the subsequent cellular uptake via CD44-mediated endocytosis. As a result, cancer cells were labeled with antigenic peptides presented by surface major histocompatibility complex class I molecules thus favoring elimination by CD8+ cytotoxic T lymphocytes (Figure 7).

## 4. Conclusions and Perspectives

The past two decades have seen great efforts from the scientific community in the development of effective antitumor regimes able to face the highly heterogeneous nature of cancers at the cellular and sub-cellular levels, expanding the concept of personalized medicine from the discovery of new biological targets to the optimization of the vectorization of therapeutics within the body to reduce unfavorable cross-toxicity to healthy organs and tissues. 

In this paper, we highlighted the role of HA as a targeting element as nanoparticle systems for the selective delivery of bioactive agents to cancer cells, showing the promising outcomes of using HA-SANPs. Although some promising results in both in vitro and in vivo investigations, severe limitations still hinder an effective bench to clinics translation of the proposed nanocarriers. 

Thus, for a more comprehensive analysis of such limitations, and to hypothesize key solutions to these issues, the literature data discussed in this review and shown in Table 1, Table 2, Table 3, Table 4, Table 5, Table 6 and Table 7 are summarized in the following table (Table 8).

Here, the overviewed research was initially classified into five groups based on the adopted HA derivatization route, and then the incidence in the use of each HA-derivative group for a specific cancer type was calculated as a percentage of total studies, considering that a single paper can cover more than a single cancer cell line and/or in vivo model at once. Moreover, as an indication of the complexity of the fabrication strategy, both preparation methods and the presence of a co-reactant within the nanoformulation were quantified in terms of value (%) within each group. Similarly, the presence (%) of in vitro or in vivo validation of the proposed HA-SANPs was assessed to show the progress of the research, while the stimuli responsivity, together with the choice of the loaded therapeutic, classified in terms of cytotoxic, MDR reversal, PTT, PDT, and imaging agents, allowed the potential application of each system to be quantitatively determined. 

From the analysis of report data in Table 8, it is evident that most of the HA derivatization used for the preparation of HA-SANPs involved the coupling with lipidizing (30%) and polymeric (22%) materials. Lipidized materials, indeed, are able to spontaneously reorganize in self-assembling structures in water media, thus allowing easy fabrication methods such as thin-film hydration (40%), dialysis (29%), and simple dispersion in water media (17%). On the other hand, polymeric materials offer high chemical versatility allowing for the insertion of stimuli-responsive functionalities, including redox (40%), pH (13%), and light (3%), as well as the possibility to reach a direct conjugation with the bioactive molecule in prodrug systems (25%). Prodrug HA-SANPs were widely explored as tools for improving the pharmacokinetics profile of conventional cytotoxic drugs (29%) and, more interestingly, of PDT/PTT and immunostimulatory agents. Finally, the formation of supramolecular assemblies was also reported (12%), particularly for obtaining HAase-responsive delivery vehicles (18%). Pristine HA (11%) was also useful to prepare HA-SANPs by virtue of electrostatic interactions with cationic polymers or biologically active molecules such as drugs and genes. 

As far as the investigated tumor types, breast cancers are the most studied in almost all groups, followed by lung and colon, due to both the overexpression of CD44+ receptors and the high incidence between populations. Most of the studies are well supported by investigations in in vivo models, and this can facilitate the translation to the clinics, but some key issues should be addressed.

At first, it should be considered that not all the HA-SANPs preparation routes match the requirements of clinical applications. As extensively discussed by Foulkes et al., there is currently very little regulatory guidance in the area of nanomaterials for biomedical applications, with the manufacturing process often being hit or miss for nanomaterial stability [214]. Although the self-assembly process is not the limiting step, since it is mainly based on the spontaneous insurgence of weak intermolecular forces (e.g., electrostatic attraction, hydrogen bonding, and hydrophobic modification) reducing the possibility of any toxic cross-reactivity, the multiple reaction steps often required for the synthesis of the tailored HA-derivative, cannot be easily scaled at the industrial level, and require significant modification to fit with the good manufacturing procedures rules [215]. From a therapeutic point of view, despite the key advantages of high and reproducible drug loading, site-specific vectorization, and the ability to bypass some MDR pathways (e.g., drug efflux transporters), tailoring the physicochemical properties for optimal therapeutic efficacy is still challenging, especially in the case of prodrug HA-SANPs. The conjugation of bioactive molecules to the polymeric backbone, indeed, has two opposite effects. The solubility, circulation through the bloodstream, and permeability are greatly enhanced, but the chemistry of the conjugation can compromise the particle-target interaction [216]. 

Moreover, recent trends point toward the fabrication of multifunctional HA-SANPs, where a dual targeting element and/or a penetration enhancer moiety are anchored. The different functionalities within multifunctional nanoparticles, indeed, can act synergistically to achieve maximal anti-tumoral activity [217]. 

In our opinion, only the synergistic combination of different approaches, including active targeting and stimuli responsivity, as well as the co-loading of multiple therapeutics (e.g., conventional cytotoxic drugs and PDT/PTT agents) can lead to some significant results. HA-SANPs well address these needs, and promising results were also obtained at the border between chemo- and immune-therapy, which is the new and more promising approach for cancer eradication. Deep integration between basic and industrial research is required, together with multidisciplinary synergistic expertise exchange, which can make the applicability to HA-SANPs not a chimera but an eye-catching future.

## Figures and Tables

**Table 1:** HA-SANPs obtained by electrostatic interactions.

Composition (*Preparation*)		Bioactive Agent	Performance				Outcome	Ref.
HA-Derivative	Other Components		Cancer Type	In Vitro		In Vivo		
				CD44+	CD44−			
HA	CDDP	CDDP	Lung	LLC	---	LLC Xm	Control Release (pH) Selective Biodistr	[45]
(*Water dispersion*)								
HA	CDDP/SRF	CDDP/SRF	Liver	HepG2	---	HepG2 Xm	Control Release (pH) Synergism Selective Biodistr	[46]
(*Water dispersion*)								
HA	CDDP/GFT CDDP/MTX	CDDP/GFT CDDP/MTX	Breast	MDA-MB-231	MCF-7	---	Targeting Multidrug therapy Sustained Release	[47]
(*Water dispersion)*								
HA	FCP-Tph	FCP-Tph	Breast	MDA-MB-231 4T1	NIH 3T3	S-D Rats 4T1 Xm	PDT Synergism Selective Biodistr	[48]
(*Sonication*)								
HA	PRTS-miR-34a	miR-34a	Breast	MDA-MB-231	MCF-7	MDA-MB-231 Xm	Control Release (pH) Synergism Selective Biodistr	[49]
(*Water dispersion)*								
HA/TPP	CS	miR-34a DOX	Breast	MDA-MB-231	---	MDA-MB-231 Xm	Control Release (pH) Synergism	[50]
(*Ionic crosslinking*)								
HA	CS SBE-βCD	CUR	Colon	HT-29	I407	---	Targeting Synergism	[51]
*(Ionic coordination)*								
HA-SH *	CS	DOX	Breast	SKBR3	---	---	Control Release (pH/redox)	[52]
(*Water dispersion*)								
HA-SH	NOCC	DOX CaP-siRNA	Cervix	HeLa	---	---	Controlled Release (pH/redox) Synergism	[53]
HS-HA-DA	---							
*(Ionic coordination)*			Ovary	OVCAR-3/MDR				
HA-SH ^#^-oDNA *	---	---	Cervix	HeLa	NIH-3T3	---	Cell Blebbing and Death	[54]
(*K^+^-dependent self-assembly*)								
HA	---	DOX	Bone	K7	---	S-D Rats K7 Xm	Control Release (pH) Synergism Selective Biodistr	[55]
(*Ionic crosslinking*)								
HA-His *	---	DOX/Ce6/Mn^2+^	Skin	B16	---	B16 Xm	Control Release (pH/redox) MRI/PDT/Synergism	[56]
(*Ionic crosslinking*)								
HHA	BSA	CDDP/ICG	Liver	HepG2	L929	HepG2 Xm	Control Release (redox) PTT/Synergism Selective Biodistr	[57]
(*Desolvation + * *coordination crosslinking*)								
HA	MPL/QS21/ R837	MPL/QS21/ R837/OVA	---	BMDCs	RAW 264.7	C57BL/6 BALB/c mice	Selective Biodistr Immunotherapy (OVA antigen)	[58]
(*Dialysis*)			Lymphatic system	---	---	EG7-OVA Xm		

* Carbodiimide chemistry; ^#^ NaBH_3_CN + DTT; BSA: Bovine serum albumin; CaP: Calcium phosphate; CDDP: Cisplatin; Ce6: Chlorin e6; CS: Chitosan; CUR: Curcumin; Cys: Cystamine; DA: Dopamine; DOX: Doxorubicin; FCP: Ferrocene cyclopalladated compound; GFT: Gefitinib; HA: Hyaluronic acid; HHA: Hydrazided HA; His: Histidine; ICG: Indocyanine green; MPL: 3-O-desacyl-4′-monophosphoryl lipid A; MRI: Magnetic Resonance Imaging; MTX: Methotrexate; NOCC: N,O-Carboxymethyl chitosan; oDNA: DNA oligonucleotide; OVA: Ovalbumin; PDT: Photodynamic therapy; PRTS: Protamine sulfate; R837: Imiquimod; S-D: Sprague Dawley; SBE: Sulphobutyl-ether; SRF: Sorafenib; Tph: 5,10,15,20-Tetrakis(4-aminophenyl)-porphin; TPP: Tripolyphosphate; Xm: Xenograft mice.

**Table 2:** HA-SANPs obtained by hydrophobic interactions of steroid-modified HA.

Composition (*Preparation*)		Bioactive Agent	Performance				Outcome	[Ref]
HA-Derivative	Other Components		Cancer Type	In Vitro		In Vivo		
				CD44+	CD44−			
HA-CHL *	---	2b/SiRNA	Skin	B16-F10	RAW264.7	---	Targeting Control Release (pH)	[59]
(*Sonication*)								
GE11-HA-cys-CHL *	---	DOX	Breast	MCF-7 MDA-MB-231	---	MDA-MB-231 Xm	Dual Targeting Control Release (Redox) Synergism	[60]
(*Sonication*)								
HA-cys-CHL *	---	IR780	Breast	MDA-MB-231	---	MDA-MB-231 Xm	PTT/PDT Selective Biodistr Synergism	[61]
(*Dialysis*)								
HA-CHL *	HSCP	DOX/PTX	Breast	MCF-7	L929	---	Control Release (pH) Synergism	[62]
(*Embedding*)			Liver	---	HepG2			
KLVFF-pA ^§^- HA-CHL *	LipoidS100/ CHL/ DSPE-mPEG	KLVFF DOX	Breast	4T1	HUVEC	Balb/c mice 4T1 Xm	Synergism Metastasis Inhibition	[63]
(*Thin-film hydration*)								
HA-TST *	---	CPT/DOX	Breast	MCF-7	---	---	Control Release (pH) Synergism	[64]
(*Dialysis*)								
HA-5βCA-Cy7.5 *	---	---	Breast	MDA-MB 231	---	---	Targeting Control Release (HAase)	[65]
*(Water dispersion)*			Prostate	PC-3				
HA-5βCA-Cy5.5 *	---	---	Squamous	SCC7	CV-1	SCC7 Xm	Selective Biodistr	[66]
*(Water dispersion)*								
HA-5βCA *	---	PTX	Squamous	SCC7	NIH-3T3	SCC7 Xm	Targeting Synergism Selective Biodistr	[67]
*(Sonication)*								
HA-5βCA *	---	PTX	Colon	HT29	NIH-3T3	---	Targeting Control Release (HAase) Selective Biodistr	[68]
			Lung	A549		---		
*(High-pressure homogenization)*			Breast	MDA-MB 231		---		
			Liver	HepG2		---		
			Skin	MDA-MB-435		MDA-MB-435 Xm		
HA-5βCA *	---	PFP	Blood	CL	---	---	Echogenic Diagnosis	[69]
*(O/W Emulsion)*			Colon	---		HT-29 Xm		
PEG-NH_2_-HA-5-βCA-Cy5.5 *	---	---	Squamous	SSC7	CV-1	SSC7 Xm	Selective Biodistr	[70]
			Colon	HCT116		---		
*(Water dispersion)*			Breast	MDA-MB 231		---		
PEG-NH_2_-HA-5-βCA *	---	DOX CPT	Squamous	SSC7	NIH-3T3	SSC7 Xm	Control Release (HAase) Selective Biodistr	[71]
			Colon	HCT116		---		
*(Sonication)*			Breast	MDA-MB 231		---		
PEG-NH_2_-HA-5-βCA-Cy5.5 *	---	IRT	Colon	---	---	HT-29 Xm	Diagnosis Synergism Selective Biodistr	[72]
						CT-26 Xm		
*(O/W Emulsion)*								
HA-DOCA-His *	---	PTX	Breast	MCF-7	---	MCF-7 Xm	Control Release (pH) Synergism	[73]
(*Sonication*)								
HA-cys-DOCA-His *	---	PTX	Breast	MDA-MB-231	---	MDA-MB-231 Xm	Control Release (Redox) Synergism	[74]
(*Dialysis*)								
mPEG-HA(DOCA)-NAC *	---	PTX	Breast	MCF-7	---	---	Control Release (Redox) Synergism Selective Biodistr	[75]
(*Sonication*)			Liver	---		H22 Xm		
HA-DOCA-His *	PF 127	DOX	Breast	MCF-7 MCF-7/ADR	---	MCF-7/ADR Xm	Control Release (pH) Resistance Reversal	[76]
(*Dialysis*)								

* Carbodiimide chemistry; ^§^ Click chemistry; 2b: 2b RNA-binding protein; 5-βCA: 5-β-Cholanic acid; CHL: Cholesterol; CPT: Camptothecin; Cy: Cyanine; Cys: Cystamine; DOCA: Deoxycholic acid; DOX: Doxorubicin; DSPE: 1,2-Distearoyl-sn-glycero-3-phosphocholine; GE11: targeting peptide; HA: Hyaluronic acid; HAase: Hyaluronidase; His: Histidine; HSCP: Lecithin hydrogenated; IRT: Irinotecan; KLVFF: Lys-Leu-Val-Phe-Phe peptide; mPEG: Poly(ethylene glycol) methyl ether; NAC: N-acethyl cysteine; pA: Propargylamide; PF 127: Pluronic F127; PFP: Perfluoropentane; PTX: Paclitaxel; TST: Testosterone; Xm: Xenograft mice.

**Table 3:** HA-SANPs obtained by hydrophobic interactions of lipid-modified HA.

Composition (*Preparation*)		Bioactive Agent	Performance				Outcome	Ref.
HA-Derivative	Other Components		Cancer Type	In Vitro		In Vivo		
				CD44+	CD44−			
HA-DSPE * HA-DMPE *	CHL	---	Breast	MCF-7	---	---	Biocompatibility	[77]
*(Sonication)*								
HA-DPPE ^$^	CHL/DPPC/PG	C12GEM	Pancreas	MiaPaCa2	VIT1	MiaPaCa2 Xm	Synergism Selective Biodistr	[78]
*(Thin-film hydration)*								
HA-PEG-DSPE *	Tf-PEG-DSPE * GM/DOTAP/PC	pDNA	Lung	A549	---	A549 Xm	Dual Targeting Sustained Release Enhanced Transfection	[79]
*(O/W emulsion)*								
HA-CE ^£^	---	HB PTX	Lung	A549	---	A549 Xm	Sustained Release Synergism/PDT	[80]
(*Dialysis*)								
HA-CE ^£^	DOTAP/DOPE	pDNA	Breast	MDA-MB-231	NIH-3T3	---	Synergism	[81]
*(Thin-film hydration)*								
HA-CE ^£^	PC/CHL	DOX/MGV	Breast	MDA-MB-231	---	S-D rats MDA-MB-231 Xm	Control Release (pH) Selective Biodistr Synergism/MR Imaging	[82]
*(Thin-film hydration)*								
HA-CE ^£^	P85	DTX	Brain	U87-MG	---	---	Sustained Release Synergism Resistance Reversal	[83]
*(Thin-film hydration)*			Breast	MCF-7		MCF-7/ADR		
				MCF-7/ADR				
His-HA-DDA *	---	DOX	Breast	4T1	---	4T1 Xm	Control Release (pH) Selective Biodistr Synergism	[84]
*(Dialysis)*								
HA-DDA *	Miglyol812 Tween80 SolutolHS15 CTAB	DTX	Lung	A549	---	---	Targeting Enhanced Uptake	[85]
*(Self-emulsification)*								
HA-HDA *	DPPC	IONPs/DTX	Breast	MCF-7	NIH-3T3	---	Synergism PTT Magnetic Targeting	[86]
*(Thin lipid film hydration)*								
HA-HDA *	PLGA	ZnPHC	Colon	HT-29	---	HT-29 Xm	PTT Selective Biodistribution	[87]
*(O/W emulsion)*			Lung	A549	---	---		
			Liver	---	LO2	---		
HA-DO *	CaP	ICG	Lung	A549	---	A549 Xm	Control Release (pH) PTT/PDT	[88]
*(Thin lipid film hydration)*								
HA-cys-STA *	---	DOX	Colon	HCT116	HEK293 CT-26	HCT116 Xm CT-26 Xm	Control Release (Redox) Synergism	[89]
*(Dialysis)*								
HA-AUT *	---	FITC-DEX NR	Breast	MDA-MB-468	SK-BR-3	---	Control Release (Redox) Targeting	[90]
*(Water dispersion)*								
MPEG-ss-HA-HDO *	---	PTX	Breast	MCF-7	---	---	Control Release (Redox) Synergism Selective Biodistr	[91]
*(Sonication)*			Liver	---		H22 Xm		
HA-His-MGK *	---	CUR	Squamous	---	---	SCC7 Xm	Control Release (pH) Selective Biodistr	[92]
*(Thin-film hydration)*								
HA-His-MGK *	PEG-NH_2_-CS-K *	CUR	Mesothelioma	HMM-239		HMM-239 Xm	Control Release (pH) Synergism In Vivo	[93]
*(Thin-film hydration)*								
FA-HA-MGK *	---	CUR	Lung	A549	---	---	Double Targeting Controlled Release (pH)	[94]
*(Dialysis)*			Breast	MCF-7				

* Carbodiimide chemistry; ^$^ reductive amination; ^£^ TBA mediated condensation; AUT: 11-(Aminooxy)-1-undecanethiol; C12GEM: 4-(N)-lauroyl-gemcitabine; CaP: Calcium Phosphate; CE: Ceramide; CHL: Cholesterol; CTAB: Cetyl trimethylammonium bromide; CUR: Curcumin; Cys: Cystamine; DDA: Dodecylamine; DMPE: 1,2-Dimiristoyl-sn-glycerol-3-phosphatidylethanolamine; DO: 1,2-Dioleoyl-3-amino-propane; DOPE: 1,2-Dioleoyl-sn-glycero-3-phoshphoethanolamine; DOTAP: 1,2-dioleoyl-3-trimethylammonium-propane; DOX: Doxorubicin; DPPC: 1,2-dipalmitoyl-sn-glycero-3-phosphocholine; DPPE: 1,2-Dipalmitoyl-sn-glycero-3-phosphoethanolamine; DSPE: 1,2-Distearoyl-sn-glycero-3-phosphocholine; DTX: Docetaxel; FITC-DEX: Fluorescein isothiocyanate-Dextran; GM: Glycerol monostearate; HA: Hyaluronic acid; HB: Hypocrellin B; HDA: Hexadecylamine; HDO: Hexadecanol; His: Histidine; ICG: Indocyanine green; IONPs: Iron oxide nanoparticles; MGK: Menthone 1,2-glycerol ketal; MGV: Magnevist—gadopentetate dimeglumine; MPEG: Poly(ethylene glycol) methyl ether; MR: Magnetic resonance; NR: Nile red; P85: Pluronic P85; PC: Phosphatidylcholine; pDNA: Plasmid DNA; PEG: Poly(ethylene glycol); PG: Phosphatidylglycerol; PHC: Phthalocyanine: PLGA: Poly(lactic-co-glycolic acid); PTX: Paclitaxel; S-D: Sprague Dawley; STA: Stearic acid; Tf: Transferrin; Xm: Xenograft mice.

**Table 4:** HA-SANPs obtained by hydrophobic interactions of phenyl-modified HA.

Composition (*Preparation*)		Bioactive Agent	Performance				Outcome	Ref.
HA-Derivative	Other Components		Cancer Type	In Vitro		In Vivo		
				CD44+	CD44−			
HA-PBA *	---	ICG	Breast	MDA-MB-231	2000 MS1	MDA-MB-231 Xm	Selective Biodistr	[98]
(*Dialysis)*								
HA-PBA *	ORL	ORL	Pancreas	PC-3 LNCaP	---	---	Synergism	[99]
*(Dialysis)*			Breast	MDA-MB-231				
HA-TIBA *	---	DOX	Squamous	SCC7	NIH-3T3	SCC7 Xm	Control Release (pH) Selective Biodistr Synergism/CT Imaging	[100]
(*Thin-film hydration*)								
HA-DA *-IONPs	---	HCPT	Squamous	SCC7	---	SCC7 Xm	Controlled Release (HAase) Synergism Magnetic Targeting MR Imaging	[101]
(*Water dispersion)*								
HA-cys-TPE *	---	DOX	Ovary	ES2	L929	ES2 Xm	Control Release (pH/Redox) Synergism Selective Biodistr	[102]
*(Dialysis)*			Cervix	HeLa				
HA-ss-MP *	---	DOX	Colon	HCT-116	---	BALB/C mice HCT116 Xm	Control Release (pH, redox) Synergism Selective Biodistr	[103]
*(Dialysis)*								

* Carbodiimide chemistry; Cys: Cystamine; DA: Dopamine; DOX: Doxorubicin; HA: Hyaluronic acid; HAase: Hyaluronidase; HCPT: Homocamptothecin; ICG: Indocyanine green; IONPs: Iron oxide nanoparticles; MP: 6-Mercaptopurine; ORL: Orlistat; PBA: Aminopropyl-1-pyrenebutanamide; TIBA: 2,3,5-Triiodobenzoic acid; TPE: Tetraphenylethylene; Xm: Xenograft mice.

**Table 5:** HA-SANPs obtained by HA modification with polymeric materials.

Composition (*Preparation*)		Bioactive Agent	Performance				Outcome	Ref.
HA-Derivative	Other Components		Cancer Type	In Vitro		In Vivo		
				CD44+	CD44−			
HA-BSA ^°°^	---	PTX IA C-1375	Ovary	SKOV-3	A2780	---	Targeting Synergism	[104]
*(Water Dispersion)*								
HA-ss-HSA *	---	DOX	Breast	MDA-MB-231	NIH-3T3	---	Control Release (redox) Synergism	[105]
*(Water Dispersion)*								
HA-PBLG ^££^	---	---	Breast	MCF-7	---	S-D rats	Control Release (pH) Synergism	[106]
(*Nanoprecipitation)*			Brain	---	U87			
HA-PBLG ^££^	---	Dy-700	Lung	A549	H322 H358	A549 Xm	Selective Biodistr	[107]
(*Nanoprecipitation)*						H358 Xm		
HA-PBLG ^££^	---	GFT VN	Lung	A549	H322 H358	BALB/C mice H358 Xm H322 Xm A549 Xm	Selective Biodistr	[108]
(*Nanoprecipitation)*								
HA-ss-PZLL *	---	DOX IONPs	Liver	HepG2	---	BALB/C mice	Control Release (redox) MR Imaging	[109]
(*Dialysis)*								
HA-PIPASP-Ce6 *	---	DOX Ce6	Colon	HCT-116 CT-26	CV-1	CT-26 Xm	Control Release (photochemical, pH)	[110]
(*Dialysis)*								
AcHA-PLA	---	DOX	Colon	HCT-116	---	S-D rats	Selective Biodistr	[111]
(*Dialysis)*								
HA-PLGA *	---	DOX	Colon	HCT-116	---	---	Synergism	[112]
(*Dialysis)*								
HA-PLGA *	---	DTX	Breast	MDA-MB-231	MCF-7	S-D rats MDA-MB-231 Xm	Targeting Selective Biodistr	[113]
(*Dialysis)*								
HA-PLGA *	---	PpIX	Lung	A549	---	---	Sustained release PDT Synergism	[114]
(*Dialysis)*								
HA-prop-PLA	sPLGA-LA	DTX	Lung	A549	---	A549 Xm	Control Release (redox) Synergism Selective Biodistr	[115]
(*Nanoprecipitation)*								
HA-cys-PLGA	TPGS	PTX RTV	Breast	MCF-7 MDA-MB-231	MCF-12A	---	Control Release (pH, redox) Synergism/Targeting Resistance Reversal	[116]
(*Sonication*)								
FA-HA-cys-PLGA *	---	DOX	Breast	MCF-7	---	MCF-7 Xm	Control Release (pH, redox) Synergism	[117]
(*Dialysis*)								
Tf- HA-cys-PLGA *	PVA	HSP90 AUY922	Brain	U87 P5 P5/TMZ-R	---	U87 Xm	Control Release (redox) Selective Biodistr Synergism Resistance Reversal	[118]
(*Emulsion solvent evaporation*)								
HA-PLGA *	MSC	PTX	Brain	C6	---	C6 Xm	Sustained Release Synergism Selective Biodistr	[119]
(*Endocytosis*)								
HA-cys-PCL ^§^	---	DOX IONPs	Liver	HepG2	---	---	Control Release (redox) MR Imaging	[120]
(*Dialysis*)								
HA-PCL	---	I-LIP	Liver	HepG2	CCL-13	---	Targeting Radiotherapy	[121]
(*Dialysis*)								
PCL-PEG-NH_2_-HA *	---	DTX	Breast	MDA-MB-231	NIH-3T3	---	Targeting Synergism	[122]
(*O/W emulsion solvent diffusion*)								
PDA-HA-prop-PCL ^§^	---	DOX	Squamous	SCC7	---	SCC7 Xm	Control Release (redox) Selective Biodistr	[123]
*(O/W emulsion)*								
HA-PPDSMA ^§^	---	DOX	Squamous	SCC7	---	SCC7 Xm	Control Release (redox) Selective Biodistr	[124]
(*Dialysis*)								
HA-P(TMC-DTC) ^§^	---	DTX	Breast	MDA-MB-231	L929	MDA-MB-231 Xm	Control Release (redox) Selective Biodistr	[125]
(*Dialysis*)								
HA-cys-MA *	HA-tet-GALA *	Sap	Breast	4T1	---	MDA-MB-231 Xm	Control Release (redox) Synergism Selective Biodistr	[126]
				MDA-MB-231				
(*Microfluidics click chemistry*)			Lung	A549		---		
			Liver	SMMC-7721		---		
HA–ss–PNIPAAm *	---	DOX	Lung	A549	LO2	---	Control Release (redox) Targeting Selective Biodistr	[127]
(*T-triggered self-assembly*)			Breast	---		4T1 Xm		
HA-poly(DEGMA-co-OEGMA) ^&^	---	PTX	Ovary	SKOV-3	HCT-8/E11	---	Targeting Synergism	[128]
(*T-triggered self-assembly*)								
HA-m-poly(DEGM-co-CMA) ^&^	---	PTX	Cervix	HeLa	Vero	HeLa Xm	Control Release (light) Selective Biodistr	[129]
(*T-triggered self-assembly*)								
HA-PEI *	HA-Cys * PEG-NH_2_-HA *	siRNA	Breast	MDA-MB-468	---	MDA-MB-468 Xm	Selective Biodistr Synergism	[130]
			Lung	A549/A549^DDP^	H69/H69AR	A549/A549^DDP^ Xm H69/H69AR Xm		
*(Water Dispersion)*			Skin	B16F10	---	B16F10 Xm		
			Liver	---	Hep3B	---		
HA-PEI *	PEG-NH_2_-HA *	siRNA	Lung	A549/A549^DDP^	H69/H69AR	A549/A549^DDP^ Xm H69/H69AR Xm	Selective Biodistr Synergism	[131]
HA-ODA *	PEG-NH_2_-HA *	CDDP						
*(Water Dispersion)*								
HA-BPEI *	---	siRNA	Skin	B16F10	HEK-293	---	Targeting	[132]
*(Coordination)*								
HA-βCD-OEI ^$^	pDNA	pDNA	Breast	MDA-MB-231	MCF-7	---	Synergism targeting	[133]
*(Coordination)*								

* Carbodiimide chemistry, °° Maillard, ^££^ Huisgen 1,3-dipolar cycloaddition; ^§^ Click chemistry; ^&^ Reversible addition–fragmentation chain-transfer polymerization; ^$^ reductive amination; Ac: Acetyl; BPEI: Branched polyethylenimine; BSA: Bovine serum albumin; CDDP: Cisplatin; Ce6: Chlorin e6; CMA: 6-Bromo-4-hydroxymethyl-7-coumarinyl methacrylate; cys: Cystamine; DEGM: Di(ethylene glycol)methyl ether methacrylate; DEGMA: Diethyleneglycolmethacrylate; DOX: Doxorubicin; DTX: Docetaxel; Dy-700: near infrared dye 700; FA: Folic acid; GALA: Cell penetrating peptide; GFT: Gefitinib; HA: Hyaluronic acid; HSA: Human serum albumin; IA: Imidazo acridinones; I-LIP: ^131^I-lipiodol; IONPs: Iron oxide nanoparticles; LA: Lipoic acid; MA: Methacrylic acid; MSC: Mesenchymal stem cells; ODA: Octadecylamine; OEGMA: Oligoethyleneglycolmethacrylate; OEI: Oligoethylenimine; P(TMC-DTC): Poly(trimethylene carbonate-co-dithiolane trimethylene carbonate); PBLG: Poly(γ-benzyl-L-glutamate); PCL: Poly(ε-caprolactone); PDA: 2-(Pyridyldithio)-ethylamine; pDNA: Plasmid DNA; PEG: Poly(ethylene glycol); PEI: Poly(ethylenimine); PIPASP: Poly(diisopropylaminoethyl) aspartamide; PLA: Poly(L-lactic acid); PLGA: Poly(lactic-co-glycolic acid); PNIPAAm: Poly(N-isopropylacrylamide); PPDSMA: Poly(pyridyl disulfide methacrylate); PpIX: Protoporphyrin IX; prop: Propargylamine; PTX: Paclitaxel; PVA: Poly(vinyl alcohol); PZLL: Poly(N-ε-carbobenzyloxy-L-lysine); RTV: Ritonavir; Sap: Saporin; S-D: Sprague Dawley; sPLGA: star PLGA; T: Temperature; Tet: Lysine-tetrazole; Tf: Transferrin; TPGS: D-alpha-tocopheryl poly(ethylene glycol) succinate; VNS: Vorinostat; Xm: Xenograft mice; β-CD: β-Cyclodextrin.

**Table 6:** HA-SANPs obtained by supramolecular assemblies.

Composition		Bioactive Agent	Performance				Outcome	Ref.
HA-Derivative	Other Components		Cancer Type	In Vitro		In Vivo		
				CD44+	CD44−			
HA-βCD *	CUR-OXPt *	CUR-OXPt	Pancreas	PC-3	LO2	---	Control Release (pH, Ease) Synergism	[140]
			Lung	A549				
HA-PMCD *	Ps-PTX	Ps-PTX	Ovary	SKOV-3	NIH-3T3	---	Control Release (HAase) Targeting/Synergism Imaging	[141]
HA-βCD *	Fc-CA	Fc-CA	Breast	MCF-7 4T1	NIH-3T3	4T1 Xm	Control Release (pH) CDT Selective Biodistr	[142]
HA-αCD *	G-CB[8]	G-CB[8]	Lung	A549	293T	---	PDT Targeting	[143]
HA-αCD *	Trans-G	siRNA	Lung	A549	293T	---	Control Release (UV) Synergism	[144]
HA-βCD *	Ad-Pt	Pt	Breast	MCF-7	NIH-3T3	---	Control Release (HAase) Synergism	[145]
			Ovary	SKOV-3		SKOV-3 Xm		
AHA-βCD °	Ad-ss-CPT	CPT	Liver	HepG2	---	---	Control Release (pH/redox) Synergism	[146]
			Bone	---		S180		
HA-βCD *	Ad-DOTA-Gd Ad-Cy7	Gd Cy7	Breast	MCF-7	---	---	Targeting MR Imaging NIR Imaging	[147]
			Brain	---	U87-MG			
Ad-HA *	AM-βCD	CBL	Lung	A549	---	---	Control Release (HAase) Synergism ATP Depletion	[148]
Ad-HA *	βCD-TPE ^#^	TPE DOX	Breast	MCF-7	NIH-3T3	---	Control Release (pH) Targeting	[149]
Ad-HA *	βCD-CPT *	CPT	Colon	HCT-116	NIH-3T3	---	Targeting	[150]
Ad-HA *	βCD-PEI * pDNA	pDNA	Cervix	HeLa	HeLa NIH-3T3	---	Targeting	[151]
HA-βCD *	DAE-βCD ^§^	adPy-Ru	Lung	A549	293T	---	PDT Targeting	[152]
TPhPh-HA-βCD *	PMCD-SS-CPT * adPs	CPT Ps	Lung	A549	293T	---	Control Release (redox) PDT/Targeting	[153]
HA-CE ^£^-MβCD *	---	---	Breast	MDA-MB-231	NIH-3T3 HUVEC	BALB/c mice MDA-MB-231 Xm	CHL Depletion Enhanced Apoptosis Targeting	[154]
Ad-HA *	MβCD	---	Colon	HCT-116	NIH-3T3	---	CHL Depletion Enhanced Apoptosis Targeting	[155]
Ad-HA *	FA-MβCD *	---	Colon	HCT-116	---	---	CHL Depletion Enhanced Apoptosis Targeting	[156]

* Carbodiimide chemistry; ° Shiff base formation; ^£^ TBA mediated condensation; ^§^ Click chemistry; ^#^ NaBH3CN + DTT; Ad: Adamantane; Ad-Pt: Adamplatin; adPy-Ru: adamantane-polypyridyl ruthenium; AHA: Aldehyde HA; AM-βCD: hexylimidazolium modified βCD; ATP: Adenosine triphosphate; CBL: Chlorambucil; CD: Cyclodextrin; CDT: Chemodynamic therapy; CE: Ceramide; CHL: Cholesterol; CPT: Camptothecin; CUR: Curcumin; Cy: Cyanine; DAE: Diarylethene; DOTA: Tert-butyloxycarbonyl 1,4,7,10-tetraazacyclododecane-1,4,7,10-tetraacetic acid; DOX: Doxorubicin; Ease: Esterase; FA: Folic acid; Fc-CA: Ferrocene-modified cinnamaldehyde prodrug; G-CB[8]: Cucurbit[8]uril carbazole derivative; HA: Hyaluronic acid; HAase: Hyaluronidase; MR: Magnetic Resonance; Mβ-CD: Methyl-β-cyclodextrin; NIR: Near Infrared; OXPt: Oxoplatin; pDNA: Plasmid DNA; PDT: Photodynamic therapy; PEI: poly(ethylenimine); PMCD: Permethyl-β-CD; Ps: Porphyrin; PTX: Paclitaxel; TPE: Tetraphenylethylene; TPhPh: Triphenylphosphine; Trans-G: Azobenzene-modified diphenylalanine; Xm: Xenograft mice.

**Table 7:** HA-prodrug nanoassemblies.

Composition		Bioactive Agent	Performance				Outcome	Ref.
**HA-Derivative**	**Other** **Components**		Cancer Type	In Vitro		In Vivo		
				CD44+	CD44−			
HA–PTX *	---	PTX	Liver	H22	---	H22 Xm	Targeting Selective Biodistr	[166]
*(Water dispersion)*								
HA-aa-PTX *	---	PTX	Breast	MCF-7	---	---	Control Release (pH, HAase) Synergism	[167]
*(Water dispersion)*								
HA-prop-dOG-PTX ^§^	---	PTX	Breast	MCF-7	---	MCF-7 Xm	Control Release (pH) Targeting Selective Biodistr	[168]
*(Solvent exchange)*								
DTX-GFLG-HA-ss-DD *	---	DTX	Breast	MDA-MB-231	MCF-7	MDA-MB-231 Xm	Control Release (pH, redox, protease)	[169]
*(Dialysis)*								
HA-d-DOX *	---	DOX	Breast	MDA-MB-231 MDA-MB-468LN	---	S–D rats MDA-MB-468LN Xm	Control Release (pH) Selective Biodistr	[170]
*(Water dispersion)*								
HA-cys-DOX *	---	DOX	Lung	A549	---	A549 Xm	Control Release (pH, redox) Selective Biodistr	[171]
*(Water dispersion)*								
Gal-PEG-ss-HA-ss-DOX *	---	DOX	Liver	HepG2	---	---	Control Release (pH, redox) Dual Targeting	[172]
*(Water dispersion)*								
HA-cys *-PMAA-PDMAEMA-P[VHim]NTf2-DOX *		DOX	Breast	4T1	L929	4T1 Xm	Control Release (pH, redox) Synergism	[173]
*(Dialysis)*			Colon	CT-26		---		
MTX-HA-ODA *	---	MTX/CUR	Cervix	HeLa	---	HeLa Xm	Dual targeting Control Release (pH) Synergism	[174]
*(Ultrasonication)*			Breast	MCF-7		---		
HA-cys-MTX *	---	MTX	Cervix	HeLa	NIH-3T3	HeLa Xm	Control Release (redox) Dual Targeting/Synergism Selective Biodistr	[175]
*(Water dispersion)*			Lung	A549		---		
HA-DTPA-CPT *	---	CPT	Breast	4T1	MCF-7	4T1 Xm	Control Release (redox) Synergism Selective Biodistr	[176]
*(Ultrasonication)*								
PLA-CDM-HA- DTPA-CPT *	---	CPT	Liver	HepG2	---	H22 Xm	Control Release (pH, redox) Synergism Selective Biodistribution	[177]
*(Electrospun)*								
HA-DAS *	TPGS	DAS/VES	Nasopharynge	HNE1 HNE1/DDP	---	HNE1 Xm	Control Release (pH) Resistance Reversal Synergism Selective Biodistr	[178]
*(Thin-film hydration)*								
HA-VES *	tLyP-1-TPGS^*^	VES/DTX	Pancreas	PC-3	---	PC-3 Xm	Sustained Release Synergism Selective Biodistr	[179]
*(Emulsion solvent evaporation)*			Breast	MDA-MB-231		---		
HA-VES *	TPGS	VES DOX/CUR	Breast	MCF-7 MCF-7/ADR	---	S-D rats 4T1 Xm	Control Release (pH) Resistance Reversal Synergism Selective Biodistr	[180]
*(Sonication)*								
HA-DAS *	TPGS	DAS/ROZ	Breast	MCF-7 MDA-MB-231	---	MDA-MB-231 Xm	Control Release (pH) Synergism Selective Biodistr	[181]
*(Thin-film hydration)*								
HA-VES *	---	VES/DOX	Breast	MCF-7 MCF-7/ADR	---	4T1 Xm	Control Release (pH) Resistance Reversal Synergism Selective Biodistr	[182]
*(Sonication)*			Liver	HepG2		---		
HA-VES *	---	VES DOX/CUR	Breast	MCF-7 MCF-7/ADR	---	4T1 Xm	Control Release (pH) MDR Reversal/Synergism Selective Biodistr	[183]
*(Sonication)*			Liver	HepG2		---		
HA-VES *	---	VES/DTX Anti-PD-L1	Skin	B16	---	B16 Xm	Synergism Immune-chemotherapy	[184]
*(Dialysis)*								
HA-CUR ^°°^	---	CUR/DOX	Cervix	HeLa	---	---	Control Release (pH) Synergism	[185]
*(Water dispersion)*			Kidney	786-O	293A			
			Liver	---	HepG2			
HA-QC *	---	QC/DTX	Liver	HepG2	---	HepG2 Xm	Control Release (pH) Synergism Resistance Reversal Selective Biodistribution	[186]
*(Dialysis)*								
HA-ss-EGCG ^££^	---	EGCG CDDP	Ovary	SKOV-3	HEK293T	SKOV-3 Xm	Control Release (HAase) Synergism Selective Biodistr	[187]
*(Dialysis)*			Colon	HCT-116				
HA-Ala-EGCG *	PEI	EGCG GzmB	Colon	HCT-116	---	---	Synergism	[188]
*(Water dispersion)*			Liver	---	HepG2			
HA-GCA *	---	GCA PTX	Liver	HepG2	HELF	---	Synergism Selective Biodistr	[189]
*(Dialysis)*			Skin	B16-F10		---		
			Breast	---		MDA-MB-231 Xm		
HA-GCA *	---	GCA PTX	Liver	HepG2	---	---	Synergism Selective Biodistr	[190]
*(Dialysis)*			Skin	B16-F10		---		
			Breast	---		MDA-MB-231 Xm		
HA-ATPh-IR780 *	---	IR780	Bladder	MB-49	---	MB-49 Xm	Control Release (HAase) PTT/Selective Biodistr	[191]
*(Water dispersion)*								
HA-DB *	---	DB	Colon	HCT-116	A2780	HCT-116 Xm	Targeting PDT	[192]
*(Sonication)*								
HA-Se-Se-Ce6	BSA	Ce6/CYC	Breast	4T1	---	4T1 Xm	Control Release (redox, ^1^O_2_) PDT/Synergism Selective Biodistr	[193]
*(Desolvation)*								
HA-DNB-DEA/NO **	---	DEA/NO DOX	Liver	SMMC-7721	HL-7702	SMMC-7721 Xm	ROS Generation Control Release (HAase, redox) Synergism	[194]
*(Sonication)*								
HA-CHL ^£^- BSAO *	---	BSAO	Skin	M14 M14/MDR	---	---	Resistance Reversal Synergism	[195]
*(Sonication)*								
HA-PDI ^ξ^	---	PDI	---	---	---	---	Control Release (HAase) Early Diagnosis	[196]
(C*oordination*)								
HA-OPV *	PAA/HEP/CHS	OPV	---	---	---	---	Control Release (HAase) Fluorescence Imaging	[197]
(C*oordination*)								
HA-OVA ^$^	---	OVA	Cervix	TC-1	---	TC-1 Xm	Immunotherapy	[198]
*(Water dispersion)*								
PEG-pep-HA-OVA *	---	OVA	Cervix	TC-1	---	TC-1 Xm	Control Release (MMP9) Immunotherapy	[199]
*(Dialysis)*								

* Carbodiimide chemistry; ^°°^ Radical polymerization; ^£^ TBA mediated condensation; ^££^ Nucleophilic addition; ** Aromatic Nucleophilic substitution; ^ξ^ electrostatic interaction; ^§^ Click chemistry; ^$^ reductive amination; aa: Aminoacid; Ala: Alanine; anti-PD-L1: programmed cell death ligand 1 (PD-L1) antibodies; ATPh: 4-Aminothiophenol; BSA: Bovine serum albumin; BSAO: bovine serum albumin oxidase; CDDP: Cisplatin; CDM: 2-Propionic-3-methylmaleic anhydride; Ce6: Chlorin e6; CHL: Cholesterol; CHS: Chondroitin 4-sulfate; CPT: Camptothecin; CUR: Curcumin; CYC: Cyclopamine; cys: Cystamine; d: Adipic dihydrazide; DAS: Dasatinib; DB: Diiodostyryl bodipy; DD: Glycodendron; DEA/NO: Diethylamine NONOate; DNB: 2,4-Dinitrobenzene; dOG: Dendritic oligoglycerol block copolymer; DOX: Doxorubicin; DTPA: 3,3′-Dithiodipropionic acid; DTX: Docetaxel; EGCG: Epigallocatechin-3-O-gallate; Gal: Galactosamine; GCA: Glycyrrhetinic acid; GFLG: Cell penetrating tetrapeptide; GzmB: Granzyme B; HA: Hyaluronic acid; HAase: Hyaluronidase; HEP: Heparin; MDR: Multi Drug Resistance; MMP9: Matrix metalloproteinase 9; MTX: Methotrexate; NTf2: Targeting peptide; ODA: Octadecylamine; OPV: Oligophenylenevinylene; OVA: Ovalbumin; P[VHim]: Poly(vinylimidazole); PAA: Poly(acrylic acid); PDI: Perylene diimide derivative; PDMAEMA: Poly(2-(dimethylamino)ethyl methacrylate); PDT: Photodynamic therapy; PEG: Poly(ethylene glycol); PEI: Poly(ethylenimine); Pep: MMP9 sensitive peptide; prop: Propargylamine; PTT: Photothermal therapy; PTX: Paclitaxel; QC: Quercetin; ROZ: Rosiglitazone; tLyP-1: Cell penetrating peptide; TPGS: D-α-tocopheryl poly(ethylene glycol) succinate; VES: α-Tocopheryl succinate; Xm: Xenograft mice.

**Table 8:** Outcomes of HA-SANPs for cancer therapy expressed as (%) of the reviewed studies.

HA-Derivative	Other Components	Preparation	Cancer Type	Bioactive Agent	Stimuli	In Vitro/In Vivo Success
HA (11) *	Bioactive (53) ** Polymer (27) ** Other (7) **	Water Disp (47) ** Coordination (47) ** Dialysis (6) **	Breast (38) **/Cervix (13) ** Liver (13) **/Bone (6) ** Colon (6) **/Lung (6) ** Lymphatic (6) ** Ovary (6) **/Skin (6) **	Drug (67) ** Gene (27) ** PTT/PDT (20) ** Imaging (7) ** Immuno (7) **	pH (53) ** Redox (27) **	(100) **/(60) **
HA-LIPOID (30) * βCA (19) ** FAD (19) ** CE (10) ** CHL (11) ** DOCA (10) ** PPL (7) ** Other (24) **	PPL (17) ** Polymer (10) ** Bioactive (2) ** Other (7) **	Thin Film (40) ** Dialysis (29) ** Water Disp (17) ** Emulsion (14) **	Breast (40) **/Colon (13) ** Lung (12) **/Squamous (12) ** Liver (5) **/Pancreas (3) ** Skin (3) **/Blood (2) ** Brain (2) **/Cervix (2) ** Mesothelioma (2) ** Ovary (2) **/Prostate (2) **	Drug (62) ** PTT/PDT (12) ** Imaging (10) ** Gene (7) **	pH (33) ** Redox (19) ** HAase (10) **	(92) **/(53) **
HA-POLYMER (22) * PLA/PLGA (30) ** PPEP (24) ** PACRY (20) ** PCL (13) ** PEI (13) **	Polymer (10) ** Bioactive (3) ** Other (3) **	Dialysis (37) ** Emulsion (10) ** Water Disp (17) ** Coordination (7) ** Temperature (10) ** Precipitation (13) ** Other (6) **	Breast (31) **/Lung (22) ** Liver (11) **/Brain (8) ** Colon (8) **/Ovary (6) ** Skin (6) **/Squamous (6) ** Cervix (2) **	Drug (77) ** Gene (13) ** Imaging (7) ** PTT/PDT (7) ** Radio (3) **	pH (13) ** Redox (40) ** Light (3) **	(94) **/(50) **
HA-CD (12) * HA-Ad (35) **	Bioactive (47) ** CD (41) ** Other (12) **	Host–Guest (100) **	Lung (30) **/Breast (25) ** Colon (15) **/Ovary (10) ** Bone (5) **/Cervix (5) ** Liver (5) **/Pancreas (5) **	Drug (47) ** PTT/PDT (24) ** Gene (6) ** Imaging (12) **	pH (24) ** Redox (12) ** HAase (18) ** Enzyme (6) ** Light (6) **	(95) **/(20)**
HA-Prodrug (25) *	Polymer (21) **	Water Disp (53) ** Dialysis (23) ** Coordination (6) ** Thin Film (6) ** Other (12) **	Breast (35) **/Liver (21) ** Cervix (11) **/Colon (9) ** Skin (9) **/Lung (5) ** Bladder (2) **/Kidney (2) ** Nasopharynge (2) ** Ovary (2) **/Pancreas (2) **	Drug (79) ** PTT/PDT (9) ** Immuno (9) ** Imaging (6) **	pH (47) ** Redox (26) ** HAase (15) ** Enzyme (3) **	(95) **/(62) **

* Incidence (%) to total reviewed studies; ** Incidence (%) within each group; 5-βCA: 5-β-Cholanic acid; Ad: Adamantane; CD: Cyclodextrins; CE: Ceramide; CHL: Cholesterol; DOCA: Deoxycholic acid; FAD: Fatty acid derivatives; HA: Hyaluronic acid; PRE: Precipitation; PACRY: Acrylic polymers; PCL: Poly(ε-caprolactone); PDT: Photodynamic therapy; PEI: Poly(ethylenimine); PLA: Poly(L-lactic acid); PLGA: Poly(lactic-co-glycolic acid); PPEP: Polypeptide; PPL: Phospholipids; PTT: Photothermal therapy.
